# Evaluation of a psychoneurological symptom cluster in patients with breast or digestive cancer: a longitudinal observational study

**DOI:** 10.1186/s12885-023-11799-x

**Published:** 2024-01-09

**Authors:** Charlotte Grégoire, Louise Baussard, Marie Ernst, Anh Diep, Marie-Elisabeth Faymonville, Martine Devos, Guy Jerusalem, Audrey Vanhaudenhuyse

**Affiliations:** 1 Sensation and Perception Research Group, GIGA Consciousness, University of Liège, Avenue de l’Hôpital, 1, 4000 Liège, Belgium; 2 APSY-V Laboratory, University of Nîmes, Nîmes, France; 3 Biostatistics and Research Method Center, University Hospital and University of Liège, Liège, Belgium; 4 Arsène Burny Cancerology Institute, University Hospital of Liège, Liège, Belgium; 5 Medical Oncology Department, University Hospital and University of Liège, Liège, Belgium; 6 Algology Interdisciplinary Center, University Hospital of Liège, Liège, Belgium

**Keywords:** Oncology, Symptom cluster, Network analysis, Quality of life, Cancer-related fatigue

## Abstract

**Background:**

A psychoneurological symptom cluster composed of cancer-related fatigue, emotional distress, sleep difficulties, and pain is very common among patients with cancer. Cognitive difficulties are also frequently associated with this cluster. Network analyses allow for an in-depth understanding of the relationships between symptoms in a cluster. This paper details the study protocol of a longitudinal assessment of the psychoneurological symptom cluster in two distinct cohorts: breast cancer and digestive cancer survivors, using network analyses.

**Methods:**

Over two years, the symptoms involved in the psychoneurological symptom cluster, along with other common symptoms (e.g., digestive symptoms, financial difficulties) and variables (i.e., self-compassion, coping strategies) will be assessed in two cohorts: breast cancer survivors (*N* = 240) and digestive cancer survivors (*N* = 240). Online questionnaires will be completed at baseline, then 6, 12 and 24 months later. Network analyses will be used to assess the configuration of the symptom cluster at each measurement time and in each cohort. Comparison of networks between two measurement times or between the two cohorts will also be done with network comparison tests.

**Discussion:**

This study will enable a better understanding of the relationships between common symptoms endured by patients with cancer. The results will be employed to develop more cost-effective interventions which, ultimately, will significantly improve the quality of life of patients with breast or digestive cancer.

**Trial registration:**

ClinicalTrials.gov (NCT05867966). Registered on the 27th of April 2023. url: https://classic.clinicaltrials.gov/ct2/show/NCT05867966.

## Background

The presence of one psychoneurological symptom cluster (PNSC) composed of cancer-related fatigue (CRF), sleep difficulties, emotional distress, and pain is particularly well-documented among patients with cancer [1–3]. Cognitive impairments are also frequently associated with this symptom cluster [4, 5]. CRF is a distressing, persistent, and subjective feeling of physical, emotional and/or cognitive tiredness or exhaustion, which is related to cancer or its treatment. It is not proportional to the person’s recent activity and interferes with their routine functioning [6]. This multidimensional symptom is considered as the most severe one endured by patients with cancer, having a high impact on their quality of life (QOL) [7–9]. Yet, few attention has been received from the healthcare professionals [10]. Emotional distress (i.e., anxiety and depression), sleep difficulties (i.e., generally insomnia), pain, and cognitive difficulties (e.g., concentration or memory difficulties) are also very common among these patients and negatively influence their QOL [11]. In addition to their own negative consequences, these symptoms evolve together and reinforce each other, participating resulting in the high burden endured by patients with cancer [2, 12]. The mechanisms involved in the PNSC are uncertain [13, 14], but it is known that the more symptoms the patient presents, the lower their QOL [15]. Despite their severe impact and their persistence up to years after treatment completion, these symptoms remain underdiagnosed and undertreated [16]. The configuration of the PNSC (e.g., intensity of the relationships between different symptoms, core/central symptom of the cluster) seems to vary according to the cancer diagnosis [1, 2] and to evolve over time [17, 18]. Most studies in oncology focused on a single symptom, or on several symptoms considered independently from each other [3, 11, 19, 20]. However, the high prevalence of the PNSC underlines the relevance of studying multiple symptoms and their interactions.

Network analysis is an innovative method that underlines a deeper understanding of the connections between symptoms. By assessing and visualizing symptom clusters as dynamic systems of interacting symptoms, these analyses allow to study symptoms in their full complexity [1, 2, 12, 18] and to compare patterns of clustering between distinct populations [2, 21] or measurement times [1, 18]. Core symptoms within a network are the ones with the strongest associations with the other symptoms, which may play a critical role in activating them [22]. Thus, targeting them could allow to design more cost-effective interventions to impact the whole cluster [1–3, 11, 17, 18, 20, 23]. As network analyses in symptom studies are scarce, there is no consensus regarding the core symptom of the PNSC yet, as it seems to vary according to the population studied, the phase of the cancer trajectory, and the methodology used. Depression [1, 20], CRF [2, 15, 18], distress [3, 17], or anxiety [23] have been recurrently shown to be the core symptoms in studies on patients with cancer.

**Fig. 1:**
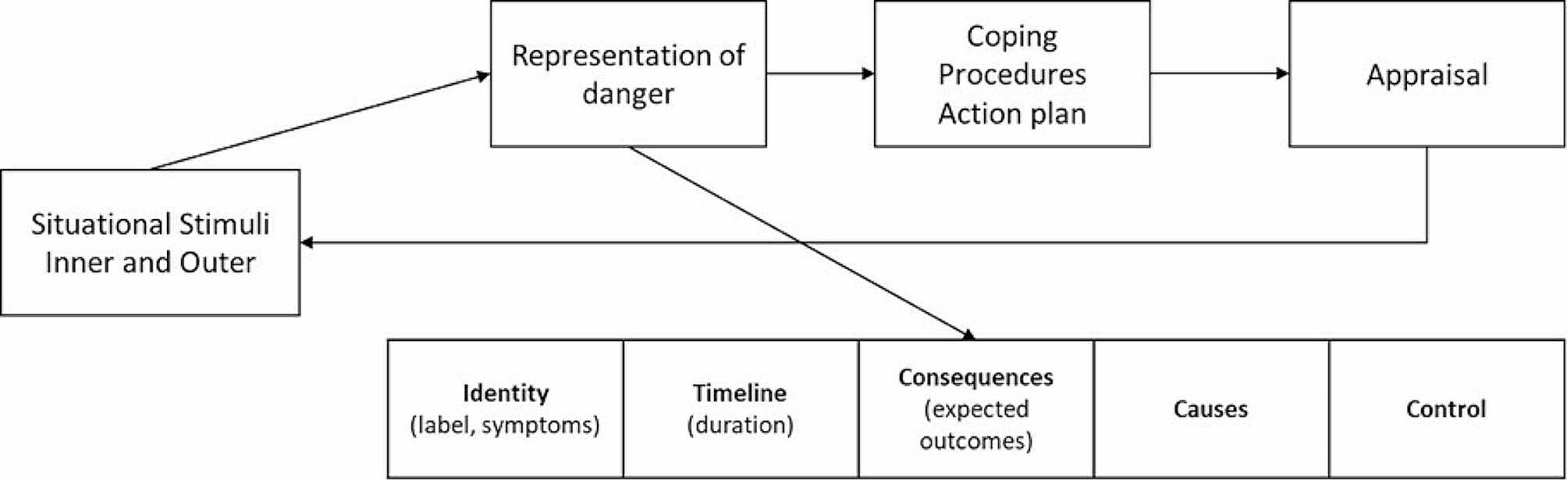
Leventhal’s common sense model of self-regulation of health, adapted from [31]

Cancer symptom management has often consisted in a “single symptom approach”, with the prescription of one specific intervention for each symptom reported by the patient, leading to complicated self-management of symptoms [11]. In the context of symptom clusters, interventions with benefits on one than more symptoms are thus highly relevant. Approaches such as mind-body techniques [11, 24–27], self-compassion learning [26–30], and cognitive-behavioural therapy [11] are relevant components to be included in such interventions. Different studies [10, 31–34] also suggested that the common-sense model of self-regulation of health and illness of Leventhal [31] (Fig. 1) could serve as a basis for developing interventions for cancer survivors, by targeting dysfunctional representations (top-down strategy) and enhancing adaptive coping (bottom-up strategy).

The studies discussed above however suffer from several limitations. First, literature on symptom clusters in oncology is heterogeneous, due to the different statistical procedures used, cancer diagnosis included, symptoms considered, and time since diagnosis. Most of them have been conducted during or shortly after treatment (i.e., generally up to one year), whereas symptoms can develop lately and persist for years after treatment [16]. Second, most of them were cross-sectional and lacked of a longitudinal evaluation of the symptom clusters [20]. Thus, there is a need for rigorous studies investigating the presence and evolution of the PNSC among distinct populations of cancer survivors [35]. There is also a critical need for cost-effective personalized interventions to alleviate these symptoms [18, 35]. Targeting a core symptom, determined for a specific population, to impact the whole PNSC seems to be of particular interest in oncology, and responds to the need for more personalized care.

## Objectives

This study will consist of a longitudinal observational study that will assess the evolution of the PNSC (i.e., CRF, emotional distress, pain, sleep difficulties) in two cohorts of patients who had cancer (women with breast cancer and patients with digestive cancer), over two years, through network analyses. Evolution of other common symptoms (e.g., cognitive difficulties, nausea/vomiting, appetite loss), QOL, coping strategies, and self-compassion will be assessed in parallel. To note, the results from this study, especially the core symptom determined in each cohort, will be used in a future study to develop a new mind-body group intervention based on the common-sense model of self-regulation of health developed by Leventhal [31]. A randomized-controlled pilot trial will then be conducted to assess its feasibility and first benefits to improve the PNSC.

## Methods

### Design and procedures

We designed a longitudinal observational study to assess the evolution of the PNSC and the respective relevant variables in two distinct cohorts of cancer survivors (breast cancer survivors and digestive cancer survivors). Questionnaires (in French; see “Statistical analyses”for more details) will be completed online (on www.alchemer.eu) at four different times: at inclusion (T1), then 6 (T2), 12 (T3) and 24 (T4) months later. Written informed consent will be obtained from each participant at the beginning of the study (included at the beginning of the online questionnaires), and the inclusion criteria are checked at the beginning of the questionnaires, so that only participants who meet the criteria will be eligible to complete them. Participants will also have the possibility to contact the principal investigators by email or phone if needed (e.g., question, adverse event, or difficulty related to the study). At each measurement time, the risk of missing responses is very low, as the online questionnaires are designed in a way that does not allow for missing answers. The short duration of the questionnaires (approximately 20 min) will also increase completion and retention. All data will be anonymized: a code will be attributed to each participant and used during the entire study. Only the principal investigators involved in this study will have access to the complete datasets. Review of the trial process and difficulties encountered are discussed regularly between the researchers involved. As no standard procedure to determine the ideal sample size for network analysis is commonly used, the number of participants required in this study will be based on other studies in oncology using similar methodology [1–3, 17, 18, 20] and including between 172 and 342 patients per cohort, and set to 200. Accounting for a drop-out rate of 10% at one year in an observational study [36], and likely a higher drop-out rate at two years, we aim to include 240 participants in each cohort.

### Eligibility

Inclusion criteria will be: ≥18-years-old, fluent in French, diagnosis of non-metastatic breast cancer or digestive cancer (i.e., anal, colorectal, gastric, oesophageal, liver, pancreatic cancers), no history of cancer and not currently in relapse, time since end of active treatments (i.e., surgery, chemotherapy, radiation therapy) < 5 years (based on the methodology and recommendations of previous studies on symptom clusters in oncology [2, 3, 35, 37]). Breast and digestive cancer were chosen because they are among the most common cancers [38]. Thus, they would be easier to recruit, and our results could be of interest for more patients, as well as their relatives and healthcare professionals. Metastatic cancers will not be included, as done in one of our previous studies [26], in order to minimize the baseline differences in the sample, as the changing nature and complexity of metastatic cancers could make it difficult to compare the results with non-metastatic cases [39].

### Recruitment

Participants will be recruited over a 2-year period. Online questionnaires will be promoted and diffused through different means: websites of the involved departments and institutions, social networks, waiting rooms of the hospital, patients’ associations, and direct contact with health professionals working with these patients, in French-speaking Belgium but also in France or other French-speaking areas.

### Assessments

Approximate duration of questionnaires completion is 20 min at each measurement time. First, socio-demographic and medical data will be collected (i.e., sex, age, marital situation, education level, professional activity, cancer diagnosis, cancer stage, personal history of cancer and treatments, possible recurrence during the study, comorbidities, neurological and psychiatric history, history of drug or alcohol abuse, previous participation in a mind-body intervention, psychotherapy, major life events). Symptoms involved in the PNSC, as well as other common symptoms and QOL, will be assessed as follows:

- *European Organization for Research and Treatment of Cancer - Quality of Life Core Questionnaire-30 (EORTC QLQ-C30)* [40]: This questionnaire (30 items) assesses the QOL of patients with cancer through 5 functional scales (physical, role, emotional, cognitive, and social functioning), three symptom scales (fatigue, nausea and vomiting, pain), a global health status/QOL scale and several single items for additional difficulties (dyspnoea, insomnia, appetite loss, constipation, diarrhoea, financial difficulties). Each item is scored on a 4-point Likert scale (‘not at all’ to ‘very much’), excepted for the global health status/QOL that is a 7-point scale (‘very bad’ to ‘excellent’). For functional scales and QOL scale, higher scores indicate better functionality and QOL. For symptom scales and single items, higher scores indicate higher symptomatology.
- *Multidimensional Fatigue Inventory (MFI-20)* [41]: This 20-item scale is designed to measure fatigue on 5 dimensions: general fatigue, physical fatigue, mental fatigue, reduced motivation and reduced activity. Suggested cut-off scores for significant general fatigue have been proposed for the general population: ≥ 11 (for men between 40 and 59 years-old), ≥ 12 (for women between 40 and 59 years-old), and ≥ 14 (for men and women older than 60 years-old) [42, 43].
- *Hospital Anxiety and Depression Scale (HADS)* [44]: This 14-item scale measures anxiety and depression, and has been validated for people with somatic illnesses. Scores between 8 and 10 on a dimension suggest the presence of anxious and/or depressive disorders, while scores between, 11 and 21 indicate the presence of such disorders.

Other variables of interest (i.e., coping strategies and self-compassion) will be assessed as follows:

- *Ways of Coping Checklist* [45, 46]: This 27-item scale assesses coping through 3 dimensions: problem-oriented coping (score range: 10–40), emotion-oriented coping (score range: 9–36), and seeking social support (score range: 8–32). Higher scores indicate higher use of specific coping strategies.
- *Self-compassion scale* [47]: This 26-item questionnaire measures 6 dimensions related to self-compassion: self-kindness, self-judgment, common humanity, isolation, mindfulness, and over-identification, with higher scores indicating higher self-compassion. A total mean score is also generated and indicates low (i.e., score between 1 and 2.49), moderate (i.e., score between 2.5 and 3.49), or high (i.e., score between 3.5 and 5) self-compassion.

### Statistical analyses

Normality of data will be checked using Shapiro-Wilk test. Sociodemographic and medical data will be described using frequencies and percentages, means and standard deviations, or medians and interquartile ranges, depending on the distribution. To estimate the networks’ structure, a standardized score (z score [1]) will be calculated for each variable or dimension. Three centrality indices will be used: strength (number and strength of the direct connections to a node/symptom, i.e., sum of absolute weights; a high strength means that the symptom is more likely to occur in conjunction with other symptoms), closeness (node’s relationship to all other nodes; closer nodes mean stronger correlation), and betweenness (importance of a node in the average pathway between other pairs of nodes; a node with a higher betweenness has greater influence in the network) [1, 2]. Symptom networks will be created for both cohorts, at each of the four measurement times. To assess the differences between two networks (i.e., between breast and digestive cancers), and over time for each cohort (i.e., between T1, T2, T3 and T4), network comparison tests will be used [1, 17]. The evolution of the other variables over time (i.e., self-compassion, self-care agency, self-care strategies already implemented to manage CRF, and the representations about CRF) will also be measured and compared through repeated measures MANOVA and post-hoc comparisons.

### Data coding and storage

The questionnaires will be completed electronically (on www.alchemer.eu) by the participants. Data encoding will be done automatically by Alchemer and will be regularly checked by one of the principal investigators. Final databases will be stored on a protected server from the University of Liège, protected by a password, only accessible to the principal investigators. Data coding and storage comply with the European General Data Protection Regulation (GDPR).

## Discussion

Recent studies highlighted the prevalence and negative consequences of the PNSC in oncology [1–3]. The core symptom of this cluster could be of major importance in the management of the cluster of symptoms as a whole [22] but it is still unclear which symptom from the cluster is the most central among patients with breast or digestive cancer. Determining this core symptom could allow to develop more cost-effective interventions, as addressing this symptom will likely decrease the other symptoms from the cluster [1–3, 11, 17, 18, 20, 23]. The development of multi-component interventions, especially those aiming to manage the whole PNSC, is then urgently needed in oncology [11]. We thus designed a longitudinal study assessing the PNSC and other symptoms in breast cancer and digestive cancer survivors, over 2 years. We hypothesize that: (1) one dimension of CRF or of emotional distress (i.e., depression or anxiety) will be the core symptom in all the obtained networks (i.e., for both cohorts and at each of the four measurement times), (2) the physical and mental dimensions of CRF will have different relationships with the other symptoms of the networks (e.g., physical fatigue could have stronger relationships with other physical symptoms such as loss of appetite or nausea/vomiting), (3) the configuration of the networks will differ according to the cohort considered (e.g., nausea/vomiting and diarrhoea/constipation could have stronger relationships with CRF, anxiety or depression in the digestive cancer cohort), and the measurement time (e.g., some correlations could weaken over time, as the intensity of some symptoms will decrease and became less important in the networks), and (4) self-compassion and coping strategies will not significantly evolve over time, even if a trend in this sense is possible due to a better adaptation to cancer.

The present study will suffer from several limitations. First, as no standard procedure to determine the ideal sample size for network analyses exist, we based our calculation on existing studies with similar methodology. It is then possible that the sample size will remain too small to allow robust results. Second, we decided to include patients who completed their active treatment within the last 5 years. One could argue that 5 years is a rather long period of time, that the psychological state and quality of life of patients are very likely to evolve during that time, and that the participants would be very different from each other, leading to an unexpected heterogeneity of our sample. However, the threshold of 5 years is commonly used in oncology studies, as it is considered to distinguish the “short-term” and “long-term” cancer survivors [2, 35, 48]. Our decision is thus in line with existing practices in the field. Finally, we will focus on two populations of cancer patients only (i.e., survivors of breast or digestive cancer). These results will then be relevant to these patients only. However, breast and digestive cancers are among the most frequent cancers [38], making our results applicable for many patients and health professionals.

Despite these limitations, this study will be one of the first to assess over a long period of time the evolution of the PNSC on two distinct populations. As described, the conduction of the present study will allow us to design a new multi-component intervention specifically targeting the core symptom determined in each cohort, and to assess its feasibility and preliminary benefits. Taken as a whole, this project is thus particularly original, timely relevant and innovative. It will bring new knowledge about the PNSC, its evolution, and its specificities according to the diagnosis, and its results will open a lot of scientific and clinical perspectives to improve symptom (self-)management of patients with cancer.

## Data Availability

The datasets used during the current study will be available from the corresponding author on reasonable request (ch.gregoire@uliege.be).
